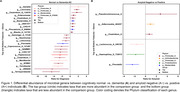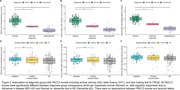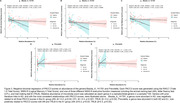# Gut microbiome features associate with cognitive scores in individuals at risk for Alzheimer’s disease

**DOI:** 10.1002/alz.085801

**Published:** 2025-01-09

**Authors:** Jea Woo Kang, Lora A. Khatib, Amanda H. Dilmore, Margo B. Heston, Tyler K. Ulland, Sterling C. Johnson, Sanjay Asthana, Cynthia M. Carlsson, Nathaniel A. Chin, Erin M. Jonaitis, Kaj Blennow, Henrik Zetterberg, Rob Knight, Rima Kaddurah‐Daouk, Federico E. Rey, Barbara B. Bendlin, Alzheimer's Gut Microbiome Project Consortium (AGMP)

**Affiliations:** ^1^ Wisconsin Alzheimer's Disease Research Center, University of Wisconsin School of Medicine and Public Health, Madison, WI USA; ^2^ University of California, San Diego, La Jolla, CA USA; ^3^ Department of Pathology and Laboratory Medicine, University of Wisconsin‐Madison, Madison, WI USA; ^4^ Wisconsin Alzheimer's Disease Research Center, Madison, WI USA; ^5^ Alzheimer's Disease Research Center, University of Wisconsin‐Madison, Madison, WI USA; ^6^ Institute of Neuroscience and Physiology, The Sahlgrenska Academy at the University of Gothenburg, Mölndal Sweden; ^7^ Department of Psychiatry and Behavioral Sciences, Duke University, Durham, NC USA; ^8^ Department of Bacteriology, University of Wisconsin‐Madison, Madison, WI USA; ^9^ Duke University, Durham, NC USA

## Abstract

**Background:**

Gut microbiome features have been linked with many diseases including Alzheimer’s disease (AD). Evidence suggests that the gut microbiota may impact cognition of AD patients. We explored the association of gut microbiota and three PACC3 cognitive scores in individuals at risk for AD.

**Method:**

Participants from the Microbiome in AD Risk Study with clinical diagnosis (N=239) and amyloid status (N=93) underwent cognitive testing and collection of microbiome samples. Three different types (Animal‐Naming Test PACC3 (AN), Category Fluency Test PACC3 (CFL), and Trail‐Making Test B PACC3 (TRLB)) of PACC scores (PACC3) were utilized followed by z‐score transformation. Fecal samples were analyzed by 16S sequencing and a taxonomic count table was generated. Kruskal‐Wallis test with Dunn's post‐hoc test was performed to find associations between PACC3 scores and group differences in diagnosis or amyloid status. Negative binomial regression (BIRDMAn, R) was performed to identify associations between microbial genus abundance and AD diagnosis, amyloid status, and PACC3 scores.

**Result:**

Several genera were differentially abundant between diagnosis groups (Normal vs. AD) and across amyloid status (A− vs. A+) (Figure 1). For example, in AD genera Clostridium_A, Bacteroides_H, and Blautia_A_141781 were more abundant, and genera Turicibacter and Prevotella were less abundant. Similarly in A+, Clostridium_A was more abundant and Prevotella was less abundant. All PACC3 scores significantly differed across diagnosis groups; however, no associations were found between PACC3 scores and amyloid status (Figure 2). Abundance of Blautia_A_141781, a genus previously linked to AD, was negatively associated with all three PACC3 scores in the A+ group (AN: β=−0.2, p=0.02; CFL: β=−0.3, p<0.001; TRLB: β=−0.2, p=0.04). Abundance of Prevotella was positively associated with PACC3 scores derived using AN and TRLB in the A+ group (AN: β=0.3, p=0.03; TRLB: β=0.3, p=0.04) (Figure 3).

**Conclusion:**

Increases in genera Blautia_A_141781 and Prevotella were associated with higher and lower cognitive performance scores, respectively. Certain microbes associate with cognitive performance in amyloid‐positive individuals. Validation of these associations in larger cohorts and diverse populations is needed.